# Evaluation of a screening program for iron overload and*HFE* mutations in 50,493 blood donors

**DOI:** 10.1007/s00277-020-04146-8

**Published:** 2020-08-26

**Authors:** Carl Eckerström, Sofia Frändberg, Lena Lyxe, Cecilia Pardi, Jan Konar

**Affiliations:** 1 Department of Immunology and Transfusion medicine Region, Sahlgrenska University Hospital, Västra Götaland, Sweden; 2 Institute of Neuroscience and Physiology, University of Gothenburg, Gothenburg, Sweden

**Keywords:** Hereditary hemochromatosis, Screening program, Blood donors, HFE

## Abstract

Early detection of individuals with hereditary hemochromatosis (HH) is important to manage iron levels and prevent future organ damage. Although the*HFE* mutations that cause most cases of HH have been identified, their geographic distribution is highly variable, and their contribution to iron overload is not fully understood. All new registered blood donors at the Sahlgrenska University hospital between 1998 and 2015 were included in the study. Donors with signs of iron overload at baseline and subsequent follow-up testing were recommended genotyping of the *HFE* gene. Of the 50,493 donors that were included in the study, 950 (1.9%) had signs of iron overload on both test occasions. Of the 840 donors with iron overload that performed *HFE* genotyping, 117 were homozygous for C282Y, and 97 were compound heterozygotes. The prevalence of C282Y homozygosity was 0.23%. Iron overload screening effectively detects individuals at risk of carrying the C282Y mutation of the *HFE* gene and enables early treatment to prevent HH complications.

## Introduction

Hereditary hemochromatosis (HH) is caused by mutations in the *HFE* gene, leading to a low production of hepcidin resulting in high uptake of iron from the intestine [1]. The subsequent iron-overload is often asymptomatic but may, left untreated, lead to liver cirrhosis, diabetes mellitus, hypothyroidism, cardiac arrhythmia and arthropathy [1]. The risk of developing sequelae is further increased by environmental factors such as excessive alcohol consumption and obesity [2].

Individuals homozygous for C282Y make up only 0.4% of the population [3], but many of them will gradually accumulate iron and eventually develop symptoms of the disease. The overwhelming majority of patients with HH are either C282Y homozygotes or C282Y/H63D compound heterozygotes. Around 70% of C282Y homozygotes have biochemical signs of iron overload, with levels between 73 and 94% reported in males and 55 and 69% in females [4–7]. However, it should be noted that these studies have used different cutoff levels for the definition of iron overload.

Early identification of individuals with HH is important, allowing for monitoring of iron levels and the application of therapeutic phlebotomy when needed to avoid further complications of the disease [8]. Presently, population screening for *HFE* mutation is not recommended due to unfavourable cost-benefit ratio [3]. Evaluations of screening approaches where risk groups with iron-overload are identified for subsequent*HFE* genotyping shows promising results [9, 10], but the variability in both prevalence and penetrance of C282Y mutations together with the relative scarcity of large iron-overload screening studies highlights the need of further studies to assess the cost-benefit of iron-overload screening for detection of individuals at risk of hereditary hemochromatosis.

Therefore, the aim of the study was to investigate the feasibility and usefulness of an iron-overload screening program to identify previously unknown*HFE* C282Y and H63y mutations in newly registered blood donors. We will also evaluate how using different cutoff levels will affect the ability of the screening program to identify *HFE* mutations.

## Materials and methods

The Sahlgrenska Iron Overload Study (SIOS) was started in 1998 with the aim of investigating causes and outcome of iron overload in blood donors. The study was approved by the local ethics committee in Gothenburg (approval number: 593-17; 170930).

**Fig. 1:**
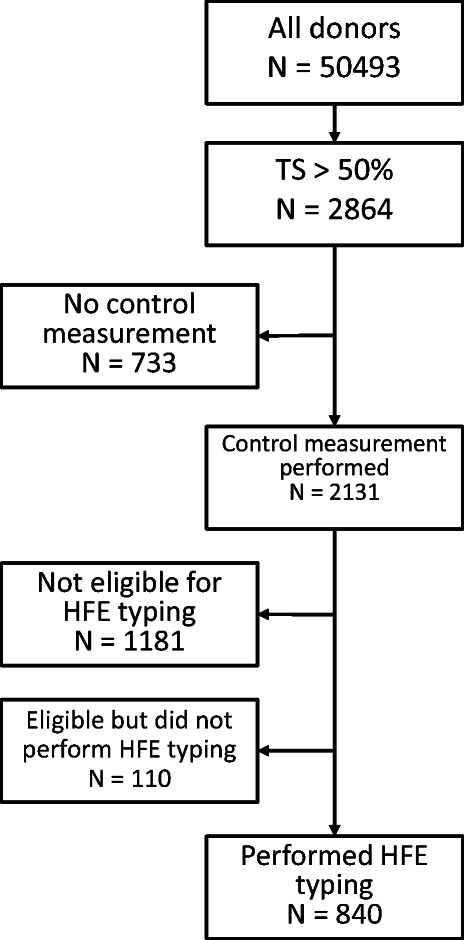
The Sahlgrenska iron-overload study screening procedure

All new registered blood donors between 1998 and 2015 that fulfilled criteria for blood donation and were not previously diagnosed with hereditary hemochromatosis or had known *HFE* mutations were included in the study. Eligibility for blood donation was established during the first visit using structured interview, checklists and blood sampling with subsequent analysis of s-Fe, s-total iron–binding capacity (TIBC) and s-ferritin. No blood was donated during the first visit. All donors that fulfilled our criteria for iron overload (transferrin saturation (TS) > 50%) were selected for subsequent control measurement of TS% and s-ferritin (μg/L). Based on the results from the control measurement, donors having TS > 50% or elevated s-ferritin (s-ferritin > 130 for men/s-ferritin > 100 for women) were recommended *HFE* genotyping. In all, 50,493 blood donors were screened, and 2864 were found to have TS > 50%. Of the donors with baseline TS > 50%, 74% (2131 donors) returned for control measurement with a mean time between baseline and control visit of 154 days. Control measurements were performed prior to blood donation. Of the 950 donors with elevated levels of TS or s-ferritin, 840 (88%) were tested for the *HFE* C282Y and H63D mutations. Levels of s-Fe, s-TIBC and s-ferritin were determined using standard laboratory methods. Figure 1 illustrates the inclusion and testing procedure.

### Genetic analyses

HFE C282Y and H63D were detected from EDTA whole-blood samples using ABI 7500 Real-Time PCR system (Applied Biosystems). Allele discrimination was performed using the ABI 7500 SDS software. Participants negative for C282Y and H63D were designated wild type.

### Statistical analyses

Patients were grouped according to *HFE* status. Demographic differences were analysed using the unpaired *t* test (age) and *χ*^2^ (sex). Levels of iron overload markers were compared between the groups with C282Y or H63D alleles and the wild type group using the unpaired *t* test. To evaluate diagnostic value for the identification of C282Y homozygotes, we calculated sensitivity/specificity, positive/negative likelihood ratio and positive/negative predictive value of different levels of TS% and s-ferritin using cross-tabulation. All analyses were performed using IBM SPSS software (version 19.0).

## Results

As can be seen in Table 1, the largest group of donors with iron-overload did not have either the C282Y or the H63D mutations. The majority were male irrespectively of *HFE* status, but the male dominance was least pronounced in the C282Y homozygous and C282Y/H63D compound heterozygous groups. These groups also have the most pronounced iron-overload compared with the wild type group.

**Table 1:** Study participants

	C282/C282	C282/H63	H63/H63	C282/WT	H63/WT	WT/WT
*N*	117	97	31	125	131	339
Age	31.1 ± 10.6	29.1 ± 10.6	28.7 ± 11.3	28.6 ± 9.8	30.8 ± 11.2	29.1 ± 9.0
Age range	18–57	18–56	18–58	18–60	18–59	18–62
Male sex %	62^**^	75^*^	84	88	82	85
TS baseline	72.5 ± 13.6^**^	60.6 ± 10.7^**^	58.4 ± 8.8	57.1 ± 7.4	57.3 ± 7.6	57.1 ± 7.4
TS F-U	67.8 ± 16.2^**^	54.8 ± 16.3^**^	51.7 ± 14.6^*^	48.7 ± 14.5^*^	46.8 ± 14.5	44.5 ± 15.1
S-ferritin	383 ± 334^**^	204 ± 186^**^	170 ± 119	140 ± 89	161 ± 106	147 ± 89

Groups carrying at least one allele of C282Y (C282) or H63D (H63) were compared with wild type donors. Values are given as mean value ± SD

*TS* Transferrin saturation %. *F-U* follow-up

*P value < 0.05 vs wild type. **P value < 0.001 vs wild type

Tables 2 and 3 shows participant characteristics divided by sex. Again, the C282Y homozygotic group have the highest levels of iron deposits, but only males in the C282Y/H63D group have elevated iron levels compared with the wild-type group. The difference between the iron levels of the C282Y homozygotic and C282Y/H63D compound heterozygotic groups and the wild type group is generally more pronounced in the follow-up testing.

**Table 2:** Iron status and*HFE* mutations for male participants

	C282/C282	C282/H63	H63/H63	C282/WT	H63/WT	WT/WT
*N*	73	73	26	101	108	287
Age	30.9 ± 10.5	28.9 ± 10.3	27.5 ± 10.1	27.7 ± 8.6	29.9 ± 11.1	28.7 ± 8.8
Age range	18–57	18–53	18–57	18–54	18–59	18–62
Baseline						
S-Fe	35.8 ± 7.3^**^	33.9 ± 6.1^*^	33.3 ± 6.7	32.0 ± 4.9	33.1 ± 5.4	32.5 ± 5.3
S-TIBC	47.1 ± 5.5^**^	55.6 ± 6.2	56.0 ± 5.2	55.8 ± 6.4	57.5 ± 6.9	57.0 ± 6.7
TS	76.0 ± 12.2^**^	61.3 ± 11.3^**^	59.4 ± 9.2	57.4 ± 7.8	57.6 ± 8.0	56.9 ± 7.7
Follow-up						
S-Fe	32.5 ± 8.3^**^	30.3 ± 9.8^**^	29.7 ± 7.9^*^	26.9 ± 8.1	26.7 ± 8.5	25.1 ± 9.1
S-TIBC	47.4 ± 6.8^**^	55.5 ± 6.4^*^	57.1 ± 5.8	55.8 ± 6.5	58.0 ± 7.0	57.4 ± 7.3
TS	69.1 ± 16.5^**^	55.0 ± 17.2^**^	51.7 ± 14.6^*^	48.4 ± 13.8^*^	46.1 ± 13.9	43.9 ± 15.0
S-ferritin	478 ± 324^**^	231 ± 147^**^	179 ± 127	150 ± 88	174 ± 110	159 ± 90

Groups carrying at least one allele of C282Y (C282) or H63D (H63) were compared with wild type donors. Values are given as mean value ± SD

*S-Fe* Serum iron. *S-TIBC* serum total iron binding capacity. *TS* transferrin saturation %. *F-U* follow-up. S-Fe and S-TIBC are reported as μmol/L, S-ferritin is reported as μg/L

*P value < 0.05 vs wild type. **P value < 0.001 vs wild type

**Table 3:** Iron status and*HFE* mutations for female participants

	C282/C282	C282/H63	H63/H63	C282/WT	H63/WT	WT/WT
*N*	44	24	5	24	23	52
Age	31.4 ± 11.0	30.1 ± 11.5	35.0 ± 16.2	32.0 ± 13.3	35.5 ± 10.8	31.1 ± 9.5
Age range	18–53	18–56	21–58	18–60	21–58	19–51
Baseline						
S-Fe	32.9 ± 7.5	32.2 ± 5.5	31.2 ± 3.9	32.0 ± 5.4	32.8 ± 6.4	32.8 ± 5.0
S-TIBC	49.6 ± 8.6^**^	55.3 ± 6.0	58.8 ± 7.0	57.2 ± 7.8	60.4 ± 10.4	57.0 ± 8.6
TS	66.8 ± 13.9^**^	58.3 ± 8.3	53.0 ± 1.6	55.9 ± 5.7	56.3 ± 5.5	57.7 ± 5.9
Follow-up						
S-Fe	31.4 ± 7.5^*^	28.9 ± 7.3	31.6 ± 15.1	28.2 ± 9.9	29.4 ± 10.6	26.9 ± 9.8
S-TIBC	48.2 ± 6.9^**^	52.3 ± 7.9^*^	61.4 ± 6.1	57.0 ± 7.0	58.5 ± 7.7	56.8 ± 9.1
TS	65.7 ± 15.7^**^	54.2 ± 13.5	52.0 ± 14.6	49.8 ± 17.6	49.9 ± 16.6	47.6 ± 15.4
S-ferritin	230 ± 294^**^	125 ± 260	124 ± 55^*^	97 ± 81	102 ± 57.3	79 ± 48

Groups carrying at least one allele of C282Y (C282) or H63D (H63) were compared with wild type donors. Values are given as mean value ± SD

*S-Fe* Serum iron. *S-TIBC* serum total iron-binding capacity. *TS* transferrin saturation %. *F-U* follow-up. S-Fe and S-TIBC are reported as μmol/L, S-ferritin is reported as μg/L

*P value < 0.05 vs wild type. **P value < 0.001 vs wild type

**Fig. 2:**
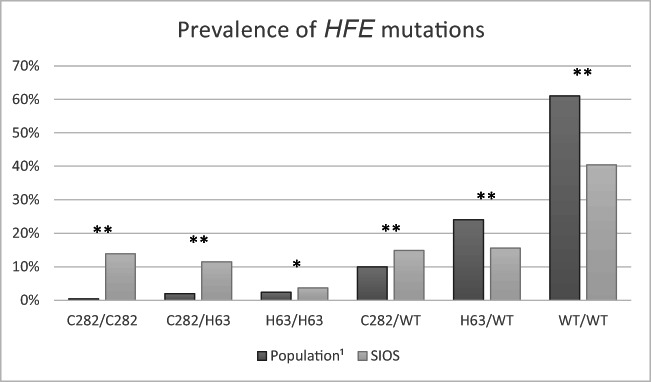
Prevalence of *HFE* mutations in the SIOS cohort compared to the general population.^1^Population prevalence based on findings in whites from the HEIRS study [11]. *χ^*2*^*P* value < 0.05. **χ^*2*^*P* value < 0.001

A comparison between the SIOS participants that fulfilled the criteria for *HFE* genotyping and the general population is displayed in Fig. 2. All groups carrying a mutation, with the exception for H63D/WT, were more prevalent in the SIOS group. The C282Y homozygous and C282Y/H63D compound heterozygous groups showed the highest overrepresentation compared with expected prevalence.

Table 4 displays a comparison of different TS% cutoff values for the discovery of C282 homozygotes. Positive likelihood ratio increased with increasing cutoff levels in both men and women with the highest levels seen for s-ferritin > 350 μg/L in men and s-ferritin > 150 μg/L in women. Defining iron overload as TS > 50% and assuming 71% penetrance of iron overload in C282 homozygotes, we performed a cross tabulation on the entire cohort resulting in high specificity and positive likelihood ratios.

**Table 4:** Comparison of different TS% cutoff values for the identification of C282 homozygotes

	Sens.	Spec.	+ LR	− LR	PPV	NPV
Men						
F-U TS% > 50	84	56	1.9 (1.7–2.2)	0.3 (0.2–0.5)	18 (16–20)	97 (95–98)
F-U TS% > 55	76	70	2.5 (2.1–3.0)	0.3 (0.2–0.5)	22 (19–26)	96 (94–98)
F-U TS% > 60	71	80	3.5 (2.8–4.4)	0.4 (0.3–0.5)	29 (24–34)	96 (94–97)
F-U s-ferritin > 130	93	36	1.5 (1.3–1.6)	0.2 (0.1–0.5)	15 (14–16)	98 (95–99)
F-U s-ferritin > 350	63	94	10 (7.2–14)	0.4 (0.3–0.5)	56 (47–64)	96 (94–97)
Women						
F-U TS% > 50	81	38	1.3 (1.1–1.6)	0.5 (0.3–0.9)	31 (27–35)	86 (76–92)
F-U TS% > 55	77	61	2.0 (1.5–2.6)	0.4 (0.2–0.7)	40 (33–46)	89 (81–93)
F-U TS% > 60	60	76	2.5 (1.7–3.7)	0.5 (0.4–0.8)	46 (37–56)	85 (79–89)
F-U s-Ferritin >100	64	59	1.5 (1.1–2.1)	0.6 (0.4–0.9)	35 (28–42)	82 (75–88)
F-U s-Ferritin >150	41	88	3.4 (1.9–6.2)	0.7 (0.5–0.9)	55 (40–68)	81 (40–68)
Whole cohort assuming 71% penetrance of iron-overload in C282 homozygotes						
Baseline TS% > 50	71	95	13 (12–14)	0.3 (0.2–0.4)	4.1 (4–5)	99 (99–100)
F-U TS% > 50	71	99	49 (44–56)	0.3 (0.2–0.4)	14 (13–15)	99 (99–100)

*F-U* Follow-up. *Sens.* sensitivity. *Spec.* specificity. *+LR* positive likelihood ratio. *–LR* negative likelihood ratio. *PPV* positive predictive value. *NPV* Negative predictive value

## Discussion

The Sahlgrenska iron-overload study successfully screened 50,493 blood donors for iron-overload and was able to identify 117 donors that were homozygous for C282Y. The screening process considerably reduced the number of donors fulfilling the criteria for *HFE* genotyping, resulting in 840 (1.7%) donors ultimately genotyped. C282Y homozygotes and C282Y/H63D compound heterozygotes were highly overrepresented in the group that was genotyped compared with previous reports on the prevalence of C282Y and H63D alleles in the general population [11].

C282Y homozygotes made up 14% of the 1.7% of the cohort that performed*HFE* typing, indicating that the screening procedure produced a group with a high number of mutation carriers. The 117 C282Y homozygotes identified correspond to a prevalence of 0.23% in the screened cohort. Although the prevalence of C282Y and H63D alleles is highly variable across geographic regions in the world [12], studies on subjects with similar ancestry as ours have reported prevalence of C282Y homozygotes between 0.30 and 0.75% [4–6, 11, 13, 14]. Applying an iron-overload penetrance of 71% in homozygotes, [4–7], results in an estimation of 165 homozygotes in the cohort corresponding to a prevalence of 0.33% which is at the lower end of previously reported values. The estimated prevalence is likely too low, possibly reflecting a lower iron-overload penetrance in our young and healthy study population.

The levels of TS% and s-ferritin differed between the groups that fulfilled the screening criteria. The highest levels were seen in the C282Y homozygotes and C282Y/H63D groups. Previous studies that have investigated iron levels and *HFE* status in the population without applying screening criteria have found similar levels of TS% and s-ferritin among C282Y homozygotes as we found in our screened group [4, 5, 11]. The other groups, however, have lower levels of TS% and s-ferritin in studies without screening criteria leading us to conclude that the applied conditions for eligibility for *HFE* genotyping in the SIOS mainly discriminate donors with *HFE* mutations less strongly linked to hemochromatosis. Further support for this conclusion can be found when comparing the composition of the genotyped group in the SIOS compared with what has been reported in the general population (Fig. 2). The C282Y homozygous group (11.5%) and the C282Y/H63D compound heterozygotic group (13.9%) were highly prevalent in our iron-overload group compared to reported prevalence in the population [4, 5, 11]. Thus we conclude that the screening process was an efficient tool to select a group of individuals where relevant *HFE* mutations can be expected to be highly overrepresented.

Finding the correct cutoff value for inclusion into a screening program is fundamental. Similar screening studies have employed varying cutoff levels, ranging between TS > 45 and TS > 55%. In retrospect, it would have been useful to have had a lower cutoff of TS > 45% in the SIOS to better evaluate the varying cutoff levels that have been used in previous studies, and also because recent findings have shown that TS > 45% may be the best cutoff point for identifying C282Y homozygotes [15], which is also reflected in recent recommendations [16]. Nonetheless, the screening method employed in the SIOS using TS > 50% yielded a group with high prevalence of C282Y homozygotes. When applying a 71% penetrance of iron-overload in C282Y homozygotes in the whole cohort, we found that the screening process resulted in high sensitivity and positive predictive values, especially so when applying a two-step approach with control measurements. However, there may be situations, such as in large population screening or where a low overall cost for the program is necessary, where a high positive predictive value is more important than a high sensitivity. In those situations, based on our findings, it may be advantageous to increase the follow-up TS cutoff to 55% in both men and women resulting in a substantial increase in specificity at the cost of a small reduction in sensitivity. It should be noted, however, that it may not be necessary to identify all C282Y homozygotes as C282Y homozygotes without signs of iron overload seem to be at low risk of developing HH complications [17]. The trend towards increased *HFE* typing in individuals without biochemical signs of iron overload [18] highlights the need for the establishment of iron overload prior to genotyping for a more favourable cost-benefit ratio.

The contribution of mutations other than C282Y homozygotes to iron overload is not fully understood. Our findings that blood donors with iron overload are more likely to be C282Y/H63D compound heterozygotes, C282Y heterozygotes or H63D homozygotes is in line with previous studies [19]. The relative low penetrance of these mutations on iron overload has not yet been determined, and the probable cause is genetic and environmental factors. Additionally, C282Y heterozygotes may carry rare mutations contributing to iron overload [20].

### Limitations

Blood donors may not be representative for the general population. Although the majority of *HFE* mutation carriers are asymptomatic and that the SIOS cohort is young (mean age 29.6) and may not have had time to develop symptoms, it is possible that signs of the disease may have discouraged some individuals from blood donation resulting in an underestimation of the prevalence of *HFE* mutation carriers. Additionally, although blood donors are required to have a good command of Swedish and minorities are believed to be underrepresented as donors, we do not record ethnic origin of blood donors which could have affected the mutation frequencies. A limitation of the present study is also the lack of data for other mutations than C282 and H63. Although the C282 *HFE* mutation is the principal cause of HH, other mutations may also give rise to iron overload [21]. Another issue with the study design is the lack of standardization of test setting. The blood for laboratory analyses were drawn when the participant registered to become a blood donor, which could have happened any time during the day. As circadian rhythms may potentially affect the results [22], it would have been preferable to standardize the blood collection reflecting this.

## Conclusions

Iron overload screening using TS% effectively identifies a population with high prevalence of C282Y and H63D mutation carriers, enabling monitoring and early treatment to prevent HH complications.
